# Self‐Reported Dementia Risk for People with Parkinson’s Disease Identifying as Sexual and Gender Minorities

**DOI:** 10.1002/alz.087824

**Published:** 2025-01-09

**Authors:** Ece Bayram, Sarah Banks

**Affiliations:** ^1^ University of California San Diego, La Jolla, CA USA; ^2^ University of California, San Diego, La Jolla, CA USA

## Abstract

**Background:**

People identifying as sexual and gender minorities (SGM) may have higher risk for subjective cognitive decline and Alzheimer’s disease, although the risk for Parkinson’s disease dementia (PDD) has not been investigated. Male sex is associated with a higher risk for PDD, it is unclear whether SGM status impacts the risk.

**Methods:**

Data were obtained from Fox Insight on April 5^th^, 2023. The analysis included people (1) with adult‐onset Parkinson’s, (2) responding to questions on sex assigned at birth, gender identity, sexual orientation, (3) with at least one available Penn Parkinson’s Daily Activities Questionnaire‐15 (PDAQ‐15), (4) without dementia at baseline, based on the first PDAQ‐15 (>43). Groups consisted of people identifying as (1) SGM with female sex assigned at birth (SGM‐F, n = 75); (2) cisgender, heterosexual women (CHW, n = 2,046); (3) SGM with male sex assigned at birth (SGM‐M, n = 84); (4) cisgender, heterosexual men (CHM, n = 2,056). Sex assigned at birth and SGM status effects on dementia likelihood during follow‐up were assessed with generalized linear mixed models.

**Results:**

Out of 159 people identifying as SGM, eight (5.0%) identified as gender minorities, 144 (90.6%) identified as sexual minorities, seven (4.4%) identified as both gender and sexual minorities. At baseline, people with female sex had better PDAQ‐15 scores than people with male sex assigned at birth; SGM‐M had the lowest scores. SGM‐M had a higher dementia likelihood compared to people not identifying as SGM. After adjusting for age, education, employment status, income, perceived discrimination level, age at Parkinson’s diagnosis, baseline PDAQ‐15 scores, that differed across groups at baseline, dementia likelihood was lower for CHW compared to people with male sex assigned at birth.

**Conclusions:**

For PDD, SGM‐M can be at a higher risk than CHM; people with female sex can have a lower risk than people with male sex assigned at birth. Socioeconomic disadvantages can alter the sex effect on PDD risk, by putting SGM‐M at a higher risk and females at a similar risk level compared to people with male sex assigned at birth, as shown in unadjusted models. Socioeconomic disadvantages should be acknowledged and addressed to support the well‐being of SGM with Parkinson’s.

